# Up-regulation of CD44 in the development of metastasis, recurrence and drug resistance of ovarian cancer

**DOI:** 10.18632/oncotarget.3220

**Published:** 2015-03-13

**Authors:** Yan Gao, Rosemary Foster, Xiaoqian Yang, Yong Feng, Jacson K. Shen, Henry J. Mankin, Francis J. Hornicek, Mansoor M. Amiji, Zhenfeng Duan

**Affiliations:** ^1^ Sarcoma Biology Laboratory, Center for Sarcoma and Connective Tissue Oncology, Massachusetts General Hospital and Harvard Medical School, Boston, MA 02114, USA; ^2^ Department of Gynecology and Obstetrics, The Third Affiliated Hospital of Zhengzhou University, Zhengzhou 450052, Henan Province, China; ^3^ Vincent Center for Reproductive Biology, Massachusetts General Hospital and Harvard Medical School, Boston, MA 02114, USA; ^4^ Department of Pharmaceutical Sciences, School of Pharmacy, Northeastern University, Boston, MA 02114, USA

**Keywords:** ovarian cancer, CD44, tissue microarray, Pgp, paclitaxel

## Abstract

The clinical significance of Cluster of Differentiation 44 (CD44) remains controversial in human ovarian cancer. The aim of this study is to evaluate the clinical significance of CD44 expression by using a unique tissue microarray, and then to determine the biological functions of CD44 in ovarian cancer. In this study, a unique ovarian cancer tissue microarray (TMA) was constructed with paired primary, metastatic, and recurrent tumor tissues from 26 individual patients. CD44 expression in TMA was assessed by immunohistochemistry. Both the metastatic and recurrent ovarian cancer tissues expressed higher level of CD44 than the patient-matched primary tumor. A significant association has been shown between CD44 expression and both the disease free survival and overall survival. A strong increase of CD44 was found in the tumor recurrence of mouse model. Finally, when CD44 was knocked down, proliferation, migration/invasion activity, and spheroid formation were significantly suppressed, while drug sensitivity was enhanced. Thus, up-regulation of CD44 represents a crucial event in the development of metastasis, recurrence, and drug resistance to current treatments in ovarian cancer. Developing strategies to target CD44 may prevent metastasis, recurrence, and drug resistance in ovarian cancer.

## INTRODUCTION

Epithelium ovarian carcinoma ranks fifth in lethal tumors among women, accounting for more deaths than any other cancer of the female genital tract. Based on the estimation of ovarian cancer for 2014 by the American Cancer Society, approximately 21,980 women will receive a new diagnosis of ovarian cancer, and near 14,270 women will succumb to this disease in the United States [1]. Conventional combinations of primary cytoreductive surgery and paclitaxel-platinum chemotherapy have not had a significant impact on overall survival of ovarian cancer in last several decades [2, 3]. The overall five-year survival rates of patients diagnosed at stage III and IV of this disease is 32% and 18%, respectively [4]. Most ovarian cancer patients who were initially responsive to standard chemotherapeutic regimens will inevitably relapse with metastasis, recurrence, and drug resistance. However, the mechanisms of development of relapsed ovarian cancer are still largely unknown.

Cluster of Differentiation 44 (CD44) is a polymorphic group of proteins, mainly owing to alternatively spliced exon transcripts in the extracellular domains [5]. The heterogeneity of the protein products vary on different cell types and growth conditions (all termed as CD44 in this study). The smallest CD44 isoform, which is also known as standard or haematopoietic isoform, is ubiquitously expressed on the membrane of most vertebrate cells [6], while other larger CD44 splice variants are merely present on limited types of epithelial tissue [7]. The CD44 transmembrane glycoprotein family mediates diverse cellular processes, which involve the regulation of growth, survival, differentiation, and motility. Despite CD44 is frequently expressed in a wide variety of epithelial malignancies, including ovarian cancer. However, the clinical significance of CD44 remains controversial. Some studies have concluded that an association between CD44 expression and poor prognosis or survival outcome [8, 9], whereas other studies did not demonstrate any correlations of these two factors [10, 11]. Few studies have investigated CD44 expression in ovarian cancer patients with long-term follow up. No previous CD44 studies have been carried out with paired primary, metastatic, and recurrent tumor tissues from each individual ovarian cancer patient. Most importantly, the effect of chemotherapy treatment on CD44 expression in ovarian cancer is also unknown. The aim of this study is to further investigate and clarify expression and function of CD44 in ovarian cancer.

## RESULTS

### Unique features of the constructed human ovarian cancer TMA

In previous studies, most reports used a TMA either from different disease stages or from various histopathological types in large patient cohort [12–15]. The TMA utilized in our current study was generated from 26 well-characterized late-stage ovarian cancer patients with long-term follow-up (Supplementary Table S1). The unique features of our TMA are that tissues are collected from paired primary, metastatic, and recurrent tumor tissues each from 26 individual patients. To our knowledge, no such human ovarian cancer tumor TMA has been constructed in former reports. Most patients were grade 3 at time of diagnosis, while there were four grade 2 patients and one grade 1 patient. All the patients were disease stage III to IV with various pathological types, including serous, transitional cell, endometroid, clear cell, undifferentiated cell, etc. The time range of DFS was between 5.3 months and 53.3 months; the shortest OS of a patient was 12 months, and the longest follow-up of a living patient is 162.3 months.

### Increased expression of CD44 in metastatic and recurrent ovarian cancer tissues, and correlated with poor clinical outcome

**Figure 1:**
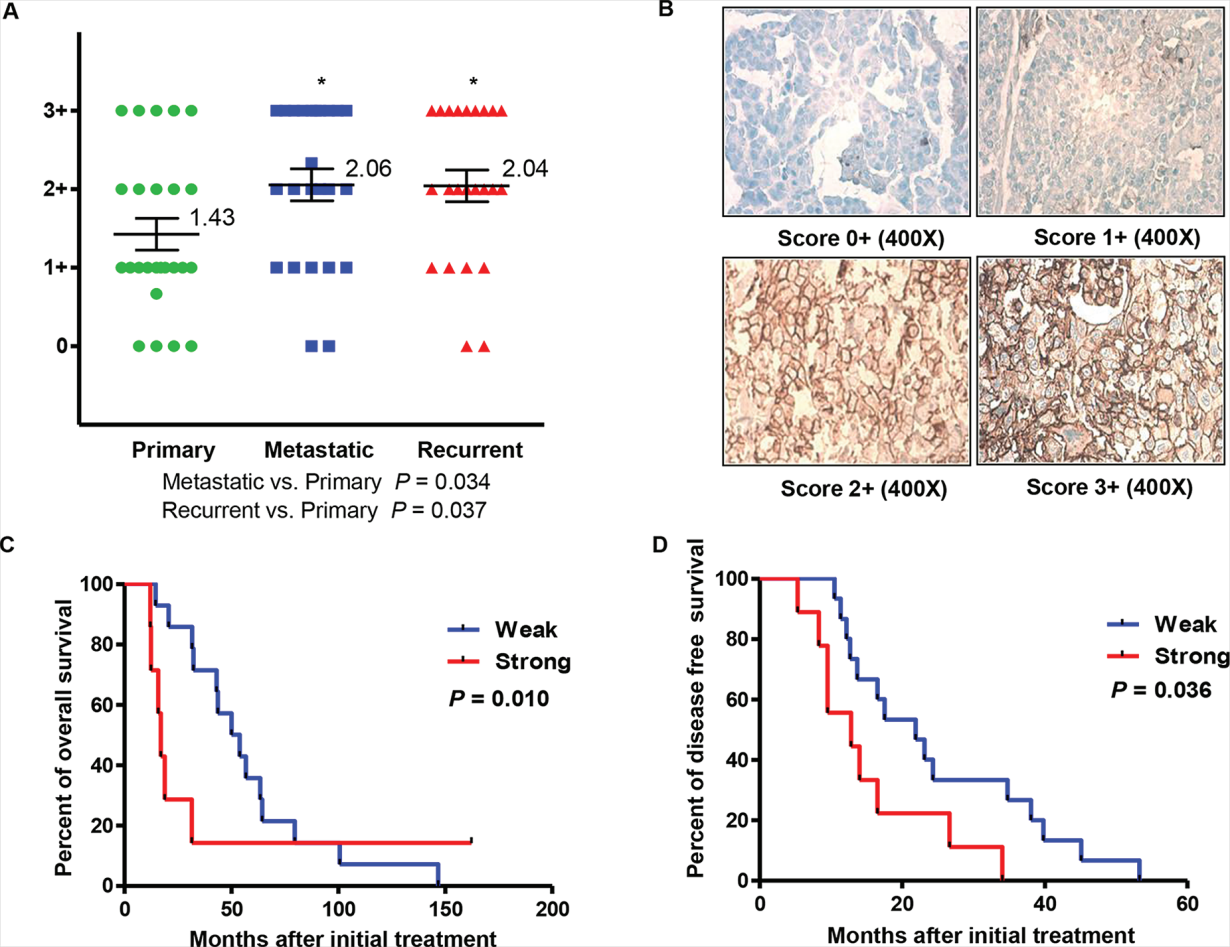
Expression of CD44 and clinical significance in primary, metastatic, and recurrent ovarian cancer Panel **(A)** distribution of CD44 immunohistochemical staining scores in primary, metastatic, and recurrent ovarian cancer. Panel **(B)** representative expression of CD44 in matched primary, metastatic, and recurrent ovarian cancers. Panel **(C and D)** correlation between expression of CD44 (CD44 staining ≤ 1+ and CD44 staining ≥ 2+) and disease free survival (Panel C) or overall survival (Panel D) in ovarian cancer patients.

Almost all of the tumor specimens presented various degrees of CD44 expression on the cell membrane (Figure 1 Panel B). Although no remarkable differences of the overall mean CD44 immunostaining scores were observed between patient-matched metastasis and recurrent ovarian cancer tumors, the expression level of CD44 was lower in primary ovarian cancer tissue than in synchronous metastasis and recurrence, with a significant difference of approximately 0.6 in staining intensity (*P* value of metastatic vs. primary = 0.034; *P* value of recurrent vs. primary = 0.037; Figure 1 Panel A). In order to clarify the initial immunohistochemistry results, follow-up analysis were performed to analyze the association of CD44 expression and ovarian cancer progression and prognosis. Patients were sorted according to CD44 expression score (weak CD44 staining score ranging from 0 to 1+, and strong CD44 staining score ranging from 2+ to 3+). A significant tendency of CD44 overexpression towards unfavorable prognosis was displayed in analysis of both overall survival and disease free survival, with *P* values at 0.010 and 0.036, respectively (Figure 1 Panel C and D).

### CD44 is overexpressed in drug resistant ovarian cancer cell lines

**Figure 2:**
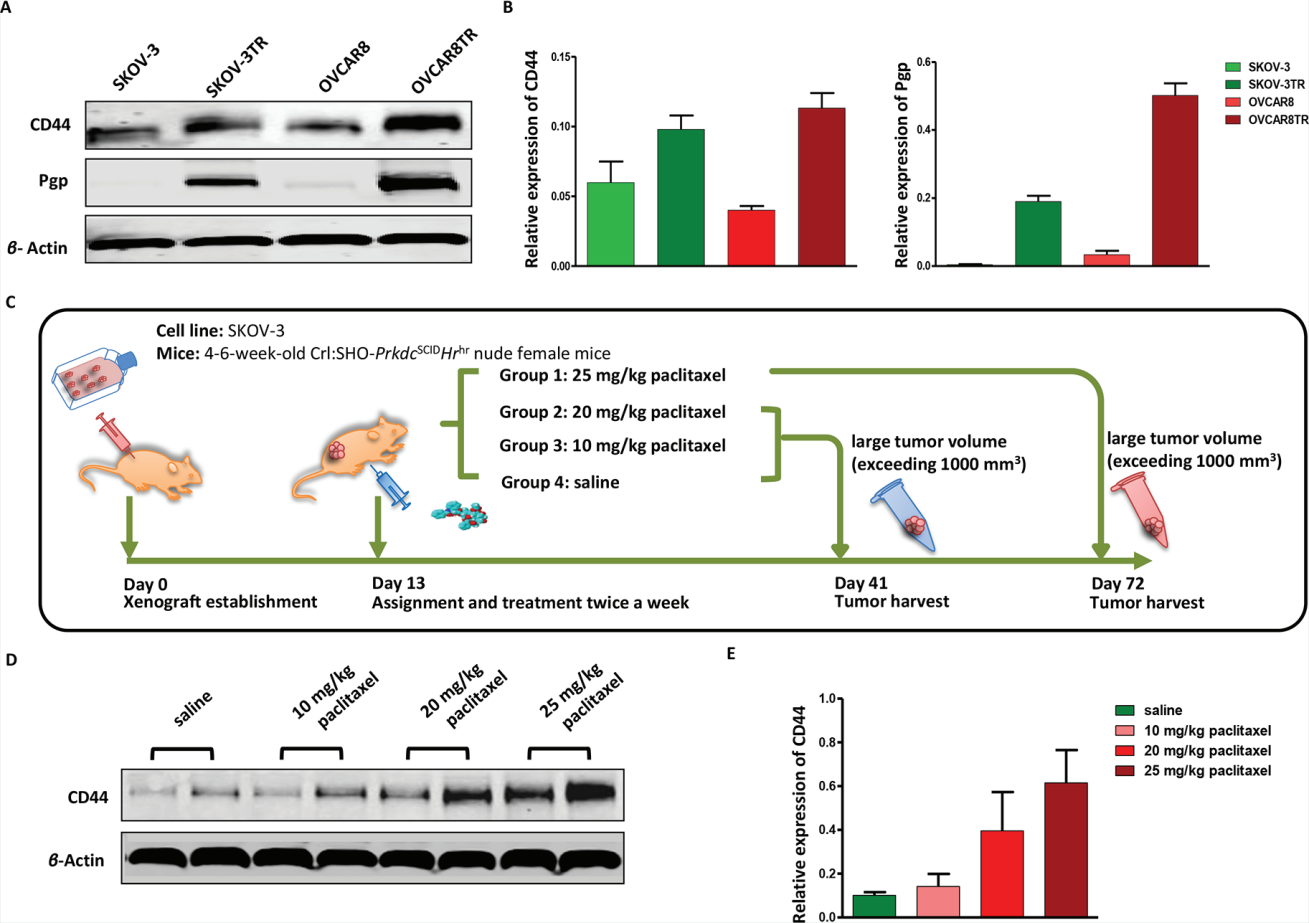
CD44 is overexpressed in drug resistant ovarian cancer cell lines, along with the appearance of tumor recurrence in ovarian cancer xenograft models Panel **(A** and **B)** relative expression levels of CD44 and Pgp in drug resistant cell lines SKOV-3TR, OVCAR8TR, and parental sensitive cell lines SKOV-3, OVCAR8 were determined by western blot. The assay was conducted in triplicate. Panel **(C)** schematic of human ovarian cancer xenograft establishment and paclitaxel treatment, each group has six female nude mice. Panel **(D)** relative CD44 protein levels in tumors of human ovarian cancer xenograft model treated with different doses of paclitaxel evaluated by western blot, which was performed in triplicate. Panel **(E)** a semi-quantitative analysis of relative CD44 protein expression determined by western bolt.

Late-stage ovarian cancer exhibits more aggressive tumor progression, especially resistant to conventional chemotherapeutic drugs. Based on the results of CD44 immunostaining in our TMA, we further examined the relative expression levels of CD44 in two pairs of well-characterized drug sensitive and resistant ovarian cancer cell lines-SKOV-3/SKOV-3TR and OVCAR8/OVCAR8TR. The western blot results demonstrated that only SKOV-3TR and OVCAR8TR exhibited strong expression of P-glycoprotein (Pgp), however both the drug sensitive and drug resistant cell lines presented a ubiquitous level of CD44 expression. Moreover, SKOV-3TR and OVCAR8TR expressed significantly higher levels of CD44 than parental sensitive cell lines (Figure 2 Panel A and B).

### Overexpression of CD44 in the tumor recurrence of human ovarian cancer xenograft model during paclitaxel treatment

Paclitaxel is the first-line chemotherapy drug in the treatment of ovarian cancer. However, little is known about the alteration of CD44 expression in recurrent ovarian cancer during chemotherapy. After an ovarian cancer xenograft model was established, the mice were treated with different regimes of paclitaxel and tumor tissues were harvested at different stages (Figure 2 Panel C). The ovarian-tumor-bearing nude mice dosed with 25 mg/kg paclitaxel were euthanized after two months, while tumor tissues from mice treated with saline, paclitaxel (10 mg/kg and 20 mg/kg) were collected after one month due to large tumor volume (exceeding 1000 mm^3^). To investigate the dynamic variations of CD44 *in vivo*, the relative protein level of CD44 was detected and analyzed by western blot and densitometry. No significant difference was observed between low-dosage (10 mg/kg) paclitaxel treated nude mice and saline treated ones; however, there is a slight increase of CD44 in 20 mg/kg paclitaxel treated mice, and dramatic overexpression of CD44 in mice dosed with 25 mg/kg paclitaxel. These results indicated that the expression of CD44 will increase in the tumor recurrence of ovarian cancer (Figure 2 Panel D and E).

### Knockdown of CD44 by shRNA induced the retardation of cell growth and inhibited the spheroid formation in 3-D culture of ovarian cancer cells

**Figure 3:**
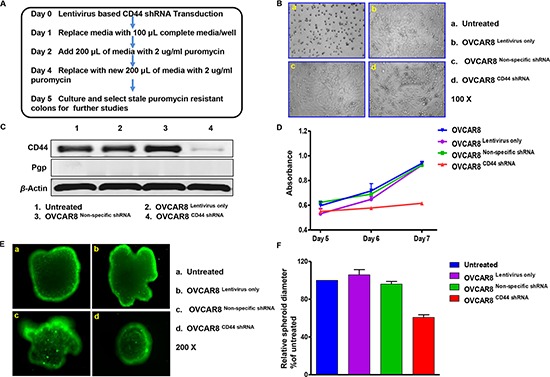
CD44 shRNA transduction suppressed sphere formation of OVCAR8 in three-dimensional culture Panel **(A)** schedule of establishing OVCAR8^Lentivirus only^, OVCAR8^Non-specific shRNA^, and OVCAR8^CD44 shRNA^ cell lines. Panel **(B)** status of cells that were successfully transduced with lentivirus and survived puromycin selection. Panel **(C)** western blot showing relative protein expression of CD44 and Pgp in established OVCAR8^Lentivirus only^, OVCAR8^Non-specific shRNA^, and OVCAR8^CD44 shRNA^ cell lines. This assay was performed in triplicate. Panel **(D)** proliferation conditions of established OVCAR8^Lentivirus only^, OVCAR8^Non-specific shRNA^, and OVCAR8^CD44 shRNA^ cell lines. The MTT assay was conducted in triplicate. **(E and F)** spheroids formation of different cells after 7-day culture and the relative diameters compared with untreated cells. The assay was conducted in duplicate.

As indicated in the western blot results, OVCAR8 expresses CD44. In order to investigate the biology of CD44 in ovarian cancer, we stably transduced ovarian cancer OVCAR8 cells by a lentivirus-mediated gene transfer system expressing CD44 shRNA. The controls were the cells without any treatments and the cells transfected with an empty vector or lentivirus-based non-specific shRNA. During the treatment period, it was observed that nearly all the untreated OVCAR8 cells were killed by 2 *μ*g/ml puromycin on Day 5, whereas there was prominent cell survival in empty vector and lentivirus-based non-specific shRNA treated wells, and half the cells transduced with lentivirus-based CD44 shRNA were still alive (Figure 3 Panel B). We then selected and sub-cultured the remaining viable cells in all the microplate wells, except for the blank control wells. Total protein was extracted to verify transduction efficiency; protein of OVCAR8 cells cultured in regular medium without any treatment was used as the control. As expected, no any alterations of CD44 were presented in OVCAR8^Lentivirus only^ and OVCAR8^Non-specific shRNA^ cells in comparison with the control, whereas the protein level of CD44 was remarkably suppressed in OVCAR8^CD44 shRNA^ cells. Moreover, none of the cell lines presented the expression of Pgp (Figure 3 Panel C). Hence, all the three cell lines (OVCAR8^Lentivirus only^, OVCAR8^Non-specific shRNA^, and OVCAR8^CD44 shRNA^) were successfully established and well characterized. Next, proliferation of the aforementioned four cell lines was assessed by MTT assay. There was obvious growth inhibition in OVCAR8^CD44 shRNA^ and no significant difference in cell growth among other control cell lines (OVCAR8, OVCAR8^Lentivirus only^, and OVCAR8^Non-specific shRNA^) (Figure 3 Panel D). Furthermore, after 7-day culture in 3-D environment, which mimics *in vivo* settings, the diameter of spheroids formed by OVCAR8^CD44 shRNA^ was relatively smaller than other cell spheroids (Figure 3 Panel E and F). These results suggested that CD44 enhances the development and progression of ovarian cancer cells.

### Knockdown of CD44 by shRNA increased the drug sensitivity in ovarian cancer cells

**Figure 4:**
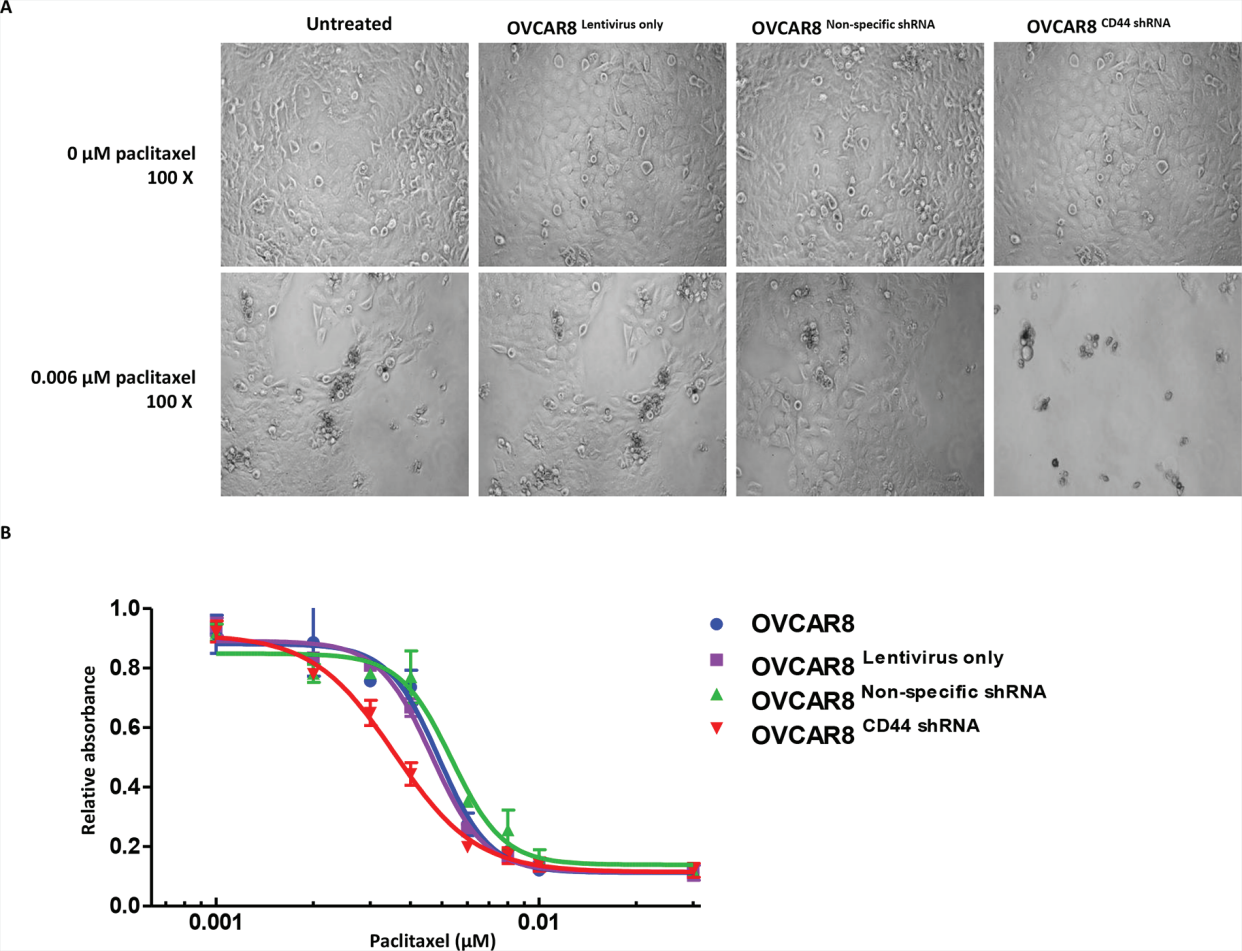
Knockdown of CD44 by lentiviral shRNA increased the paclitaxel sensitivity of ovarian cancer cells Panel **(A)** status of OVCAR8, OVCAR8^Lentivirus only^, OVCAR8^Non-specific shRNA^, and OVCAR8^CD44 shRNA^ cells that differed between representative concentrations of paclitaxel (0 *μ*M and 0.006 *μ*M). Panel **(B)** MTT assay showing cell viability of the above mention four cell lines with different concentrations of paclitaxel. The MTT assay was conducted in triplicate.

To examine whether repression of CD44 levels would increase the drug sensitivity of ovarian cancer cells, the MTT assay was performed on the established cell lines OVCAR8^Lentivirus only^, OVCAR8^Non-specific shRNA^, and OVCAR8^CD44 shRNA^ after incubating with paclitaxel. OVCAR8 cells without any treatment were used as the control. The representative pictures of different cells dosed with 0 *μ*M paclitaxel and 0.006 *μ*M paclitaxel were shown in Figure 4 Panel A. The results showed minimal survival of OVCAR8^CD44 shRNA^ in medium containing 0.006 *μ*M paclitaxel, while a substantial number of OVCAR8, OVCAR8^Lentivirus only^, and OVCAR8^Non-specific shRNA^ cells were still able to tolerate this level of paclitaxel exposure. Further analysis of the MTT data indicated that knockdown of CD44 significantly restored the sensitivity to chemotherapeutic drug paclitaxel in OVCAR8 (Figure 4 Panel B).

### Knockdown of CD44 by esiRNA in ovarian cancer cells

**Figure 5:**
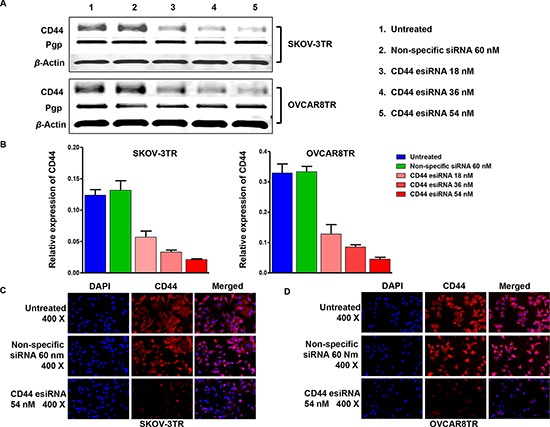
Inhibition of CD44 expression by transfection of CD44 esiRNA in ovarian cancer cell lines Panel **(A and B)** western blot demonstrating knockdown of CD44 and relative expression of Pgp in drug resistant cell lines post transfection CD44 esiRNA 48 hours. The western bolt was performed in triplicate. Panel **(C and D)** immunofluorescence photos for CD44 (red) and nuclei (blue) of ovarian cancer cell lines treated with CD44 esiRNA and non-specific siRNA. This assay was repeated twice.

Drug resistant ovarian cancer is more intractable in the clinical environment. CD44 is overexpressed in drug resistant ovarian cancer cell lines (Figure 2 Panel A and B); therefore, we examined whether disruption of CD44 expression would influence the function of SKOV-3TR and OVCAR8TR. Western blot was conducted to detect the efficacy of CD44 esiRNA transfection *in vitro*. It was found that CD44 esiRNA transfected cells showed significantly reduced protein levels of CD44, compared with untreated cells and cells dosed with non-specific siRNA (Figure 5 Panel A). Moreover, the level of CD44 expression was suppressed by CD44 targeted esiRNA in a dose dependent manner, as determined by densitometry quantification. Specifically, when normalized to untreated cells, the expression levels of CD44 in cells transfected with 18 nM, 36 nM and 54 nM CD44 esiRNA were decreased to 53.3%, 29.2%, and 18.3% for SKOV-3TR, and 48.7%, 29.2%, and 13.1% for OVCA8TR. However, knocking down of CD44 has no significant influence on Pgp expression (Figure 5 Panel B). In addition, results from the immunofluorescence study further showed that CD44 were effectively inhibited by 54 nM CD44 esiRNA in both SKOV-3TR (Figure 5 Panel C) and OVCAR8TR (Figure 5 Panel D) cells. Red color represents CD44 protein and blue color represents cell nuclei.

### Knockdown of CD44 by esiRNA suppressed the mobility and invasion activity of ovarian cancer cells

**Figure 6:**
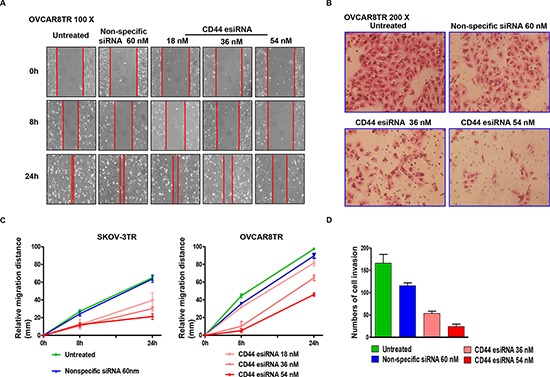
Transfection of CD44 esiRNA suppressed the migratory and invasion activity of ovarian cancer cell lines Panel **(A and C)** relative migration distance of SKOV-3TR and OVCAR8TR at different time points (0 hour, 8 hours, 24 hours) when transfected with different concentrations of CD44 esiRNA and non-specific siRNA. The wound healing assay was conducted in duplicate. Panel **(B and D)** numbers of cell invasion through the Matrigel after 24 hours when transfected with different concentrations of CD44 esiRNA and non-specific siRNA. The matrigel invasion assay was repeated twice.

Migration and invasion are crucial prerequisites for ovarian cancer metastasis and recurrence. After the confirmation of remarkable knockdown of CD44 by esiRNA in SKOV-3TR and OVCAR8TR, we next evaluated whether CD44 participates in the modulation of cell mobility. As observed in the wound healing assay, wounds were almost recovered after 24-hour migration in blank control and non-specific siRNA treated cells. However, the wound healing showed a substantial inhibition when the cells were subjected to CD44 esiRNA, especially at higher concentrations. During the 24-hour incubation, the relative migratory distances of SKOV-3TR cells transfected with 36 nM and 54 nM CD44 esiRNA were 32.67 mm and 22.15 mm, respectively; the OVCAR8TR cells dosed with 36 nM and 54 nM CD44 esiRNA migrated merely 61.54 mm and 45.19 mm (Figure 6 Panel A and B). To assess whether the invasive potential of drug resistant ovarian cancer cells changed *in vitro* when lacking CD44, transwell invasion assays were carried out after transfection using different concentrations of CD44 esiRNA. The average numbers of CD44 esiRNA (36 nM and 54 nM) treated OVCAR8TR cells invading through the matrigel were significantly lower in contrast with the blank control and the non-specific siRNA groups (Figure 6 Panel C and D). Taken together, wound healing and transwell invasion assays both demonstrated that down regulation of CD44 inhibited the migration and invasion capabilities of SKOV-3TR and OVCAR8TR cells.

## DISCUSSION

In the current study, we provided a comprehensive overall expression profile of CD44 in ovarian cancer, from individual patients’ clinical primary tumor tissue to metastasis and recurrence, and from tumor cell lines to xenograft mouse model. Our study demonstrated that both the metastatic and recurrent ovarian cancer tissues expressed higher levels of CD44 than patient-matched primary tumor samples. A pronounced association between the expression of CD44 and disease-specific survival and overall survival was observed through TMA immunohistochemistry analysis. When CD44 was stably inhibited by its specific shRNA, the proliferation speed and spheroids formation of ovarian cancer cells was inhibited under 3-D culture conditions. We also found that knocking down CD44 impaired the migratory and invasive functions. In addition, CD44 knockdown ovarian cancer cells increased sensitivity to the anticancer drug paclitaxel. Results from drug sensitive and resistant cell lines and from xenograft mouse model of ovarian cancer indicated that up-regulation of CD44 is a critical event during the progression of ovarian cancer.

The development of TMA technology introduces a cost-effective and efficient technique in conducting research. A large number of tumor samples can be processed simultaneously under uniform immunohistochemical experimental conditions [16]. This platform has facilitated evaluating the correlations between molecular variations and clinicopathological characteristics of tumors in a vast number of publications so far, including ovarian cancer. Various human ovarian cancer TMAs have been constructed in previous investigations. For instance, TMAs containing: 136 cases of epithelial ovarian carcinoma (108 high-grade serous carcinoma and 28 clear cell carcinoma) [12]; a larger patient cohort (serous 357, endometrioid 37, mucinous 88, clear cell 14, etc.) [13]; 144 formaldehyde fixed-paraffin embedded primary ovarian tumors (grade 1 to 3, FIGO stage I to IV) [14]; and 99 primary epithelial ovarian cancers, 22 peritoneal metastasis, and 13 normal ovarian samples [15]. To the best of our knowledge, no former studies have utilized the tumor TMA as what we used in this study, which was generated by 26 well characterized late-stage ovarian cancer patients with long-term follow-up. The particularly remarkable feature of this TMA is that each patient possessed a primary tumor, a synchronous metastasis, and a metachronous recurrence obtained at the time of tumor progression after initial surgery and specific platinum- and/or paclitaxel-based chemotherapy. The metastatic and recurrent tumor sites disseminated a wide spectrum of organs, such as ovary, sigmoid, liver, brain, omentum, etc. Epithelial ovarian cancers are a particularly heterogeneous type of tumor, in part due to their large size and different histological subtypes [17, 18]. In this regard, three core biopsies from morphologically representative areas of each paraffin-embedded tumor tissue block were identified and collected to minimize the variability. Moreover, it is worth mentioning that we have previously applied this TMA to prove the hypothesis that expression of pStat3 and inflammatory infiltration increased in recurrent and metastatic ovarian cancer tissues [19]. Despite the limited numbers of cases, the results deriving from this unique human TMA will provide more valuable information.

As a multifaceted and multifunctional molecule, CD44 has attracted substantial interest since its first description [20]. CD44 is known to regulate several essential cellular and molecular events, including organ development, neuronal axon guidance, numerous immune functions, haematopoiesis, and pathological processes [21–23]. Induction of CD44 expression has been addressed during the progression of ovarian cancer. However, the role of CD44 expression in epithelial ovarian cancer has not been clarified and the clinical significance of CD44 in ovarian cancer remains controversial [8–11]. On the basis of immunohistochemistry staining of our unique TMA, we found that the expression of CD44 was more pronounced in the recurrent and metastatic ovarian cancer tissues, when compared with its primary counterparts. There was a significant correlation between CD44 expression and disease free survival and overall survival. Consequently, our investigations demonstrated that high expression of CD44 predicts an unfavorable prognosis in ovarian carcinoma.

Among the critical characteristics of an aggressive malignant ovarian cancer phenotype are rapid cell proliferation, migration, and invasion. CD44 plays an important role in communication of cell-matrix interactions, cell motility, matrix degradation, proliferation, and survival. Previous reports have found that suppressing CD44 by its specific siRNA dramatically decreases the migratory potentials and invasiveness of ovarian cancer cells [24–26]. We have similar findings when knocking down CD44 by efficient and specific esiRNA in malignant drug resistant ovarian cancer cell lines. More importantly, stable CD44 knockdown ovarian cancer cell lines and their negative controls have been well established, characterized, and intensively studied in the current work. Those cell lines were cultured in 3-D environment, which can mimic *in vivo* growth conditions. When CD44 was stably knocked down by targeted shRNA, the formation of spheroids under 3-D culture and the growth speed were significantly suppressed. In recent *in vivo* reports, there was effective tumor shrinkage in xenograft models by suppressing CD44 mRNA and protein [24, 27]. It has been demonstrated that peritoneal cells produce several extracellular matrix molecules that interact with CD44 receptor, such as hyaluronic acid (HA, hyaluronan, a linear polymer of repeating disaccharide units [D-glucuronic acid (1-β-3) N-acetyl-D-glucosamine (1-β-4)]_n_), collagen, osteopontin (OPN), and L-selectin and E-selectin, etc [28–31]. The principal molecule among these is HA; when CD44 on the surface of ovarian cancer cells binds to HA on mesothelial cells, this combination may trigger peritoneal metastasis [32, 33]. A study focused on colon cancer has also demonstrated that CD44 expressed on the cell surface can facilitate binding endothelial P- or L-selectin and increase haematogenous spread of tumor [34]. Although the role CD44 expression plays in ovarian cancer recurrence, metastasis, and drug resistance is unknown, the results showing that knockdown of CD44 inhibits tumor cell proliferation and migration/invasion provides evidence that CD44 may directly participate in ovarian cancer progression.

Tumor recurrence under common cytotoxic chemotherapy is one of the most challenging problems in cancer treatment, not limited to ovarian cancer. Nevertheless, the molecular mechanisms elucidating tumor recurrence and chemoresistance are widely investigated but still poorly understood. In this study, overexpression of CD44 was found in the tumor recurrence of xenograft mouse model undergoing paclitaxel treatment. The present work, to our knowledge, is the first evaluation of CD44 expression during paclitaxel treatment *in vivo*. Moreover, the expression level of CD44 was also examined in paclitaxel-resistant cell lines. The expression of CD44 was significantly higher in the paclitaxel-resistant cells lines than in the drug sensitive parental cell lines. Previous studies have shown that reducing the expression level of CD44 by siRNA increased sensitivity to doxorubicin in breast cancer [35]. Downregulation of CD44 by miR-199a-3p significantly increased the chemosensitivity of ovarian cancer cells to cisplatin, pacitaxel, and adriamycin [27]. In accordance with the previous studies, we demonstrated that stable suppression of CD44 by lentivirus-based CD44 shRNA increased sensitivity to paclitaxel. Overexpression of MDR1 protein Pgp acts as an energy-dependent drug efflux pump to pump out many structurally unrelated chemotherapeutic drugs. It has been recognized as one of the well characterized mechanism for resistant to drug, including paclitaxel [36, 37]. Importantly, either transient or stable knockdown of CD44 cannot induce the alteration of Pgp in drug resistant or sensitive phenotypes. Although the mechanism by which CD44 induces drug resistance and tumor recurrence is unknown, our findings indicate that this does not appear to be via MDR1-dependent mechanism. Hedgehog signaling pathway has been shown to be important in the maintenance of chemotherapy resistance in a subgroup of CD44+ gastric cancer cells [38]. Overexpression of CD44 has also been shown to promote resistance to etoposide-induced apoptosis by alteration of levels of caspase 9, caspase 3, Bcl-xl, and Bak, down-regulation of pRB, and phosphorylation of AKT in colon cancer [39]. Genetic loss of CD44 in murine chronic lymphocytic leukemia had a negative impact on the phosphoactivation of important antiapoptotic regulators, ERK1/2 and AKT kinases [40]. Another potential mechanism is that CD44 functions in drug resistance as an antiapoptotic factor through up-regulation of Bcl-xL in breast cancer [41].

In conclusion, we demonstrate CD44 is significantly up-regulated during the progression of human ovarian cancer, including recurrence, metastasis, and acquisition of drug resistance. CD44 plays crucial roles in ovarian cancer cell growth, migration, and invasion. These results suggest that developing new strategies to target CD44 in ovarian cancer may prevent recurrence, metastasis, and drug resistance, and improve the clinical outcome of ovarian cancer patients.

## MATERIALS AND METHODS

### Ovarian cancer tissue microarray (TMA)

A total of 26 individual ovarian cancer patients during their treatment at Massachusetts General Hospital were recruited in this study. The archived, formalin-fixed, paraffin-embedded tumor specimens from patients were selected for construction of the human ovarian cancer TMA, which was generated by the Tissue Microarray Core at the Dana-Farber/Harvard Cancer Center. Each of these 26 patients’ tumor tissue blocks were composed of: 1) a primary tumor, 2) a synchronous metastasis obtained at the time of the primary surgery, and 3) a metachronous recurrence from the same patient collected at the time of tumor recurrence after platinum- and taxane-based chemotherapy treatment. Haematoxylin and eosin-stained slides from each tissue block were read by a senior consultant pathologist, together with pathology reports to obtain representative triplicate 0.5-mm-diameter core biopsies of primary, metastasis, and recurrent ovarian cancer tumors (absence of necrosis, poorly differentiated tumor areas). Pathological identification of diagnosis and staging of all the patients was in accordance with World Health Organization criteria and the Federation International of Gynecology and Obstetrics (FIGO) guidelines on the management of ovarian cancer [42–44]. Other relevant clinical information was also collected, including ascites present at surgery, disease free survival (DFS) as the interval between date of diagnosis and date of recurrence, overall survival (OS) as the interval from date of surgery to death or to last follow-up (for censored events), as well as current patient status. The process of case collection was approved by the Institutional Review Board at Massachusetts General Hospital.

### Immunohistochemistry

The expression level of CD44 was determined based on the Immunohistochemistry Protocol (Paraffin) from Cell Signaling Technology (Beverly, MA). Briefly, 5-μm paraffin tissue section slides were baked at 60°C for 1 hour, deparaffinized in xylene for 10 minutes, and then transferred through graded ethanol for rehydration. Following the process of antigen retrieval, endogenous peroxidase activity was quenched by incubation in 3% hydrogen peroxide. After protein blocking with blocking solution (Cell Signaling Technology) for 1 hour at room temperature, primary antibody was applied at 4°C overnight in a humidified chamber. Each step preceded three Tris-buffered saline (TBS) rinses, and the bounded antibody on the array was detected by using SignalStain^®^ Boost Detection Reagent (Cell Signaling Technology) and SignalStain^®^ DAB (Cell Signaling Technology). Prior to imaging, the section was counterstained with hematoxylin QS (Vector Laboratories) and mounted with VectaMount AQ (Vector Laboratories) for long-term preservation. The expression of CD44 was evaluated by two scientists who had no knowledge of the clinical data and the other viewer's score. The immunostaining intensity pattern of CD44 was assessed on a scale semiquantitatively as follows: 0, no staining; 1+, weak staining; 2+, moderate staining; and 3+, intense staining. Scoring was calculated from the mean of the two independently conducted assessments.

### Tumor xenograft model and treatment

In order to determine the effect of chemotherapy treatment on CD44 expression in ovarian cancer *in vivo*, an ovarian cancer xenograft mouse model was established and paclitaxel treatment was subsequently carried out. Specifically, SKOV-3 cell suspension (2 × 10^6^) was mixed with matrigel (BD Biosciences, San Jose, CA) in volume ratio 1: 1, and the mixture was injected into the flanks of 4–6-week-old Crl:SHO-*Prkdc*^SCID^*Hr*^hr^ nude female mice (Charles River Laboratories, Ann Arbor, MI) subcutaneously. Approval of the study protocol was obtained and supervised by Massachusetts General Hospital Subcommittee on Research Animal Care (SRAC). When the tumor volume of mice reached approximately 150 mm^3^, the mice (six per group) were randomly assigned to control or treatment groups, and dosed with saline or different dosages of paclitaxel (10 mg/kg, 20 mg/kg, 25 mg/kg, respectively) by tail vein injection. The treatment schedule was conducted twice a week until the tumor grew larger than 15 mm in diameter or exceeded 1000 mm^3^. The health of the mice was monitored daily during the treatment period. The animals were weighed and tumor size was measured by a caliper twice a week. At the end of the experiments, the animals were euthanized; tumors were harvested and stored at –80°C for protein extraction.

### Cell lines and reagents

Ovarian cancer cell lines SKOV-3, OVCAR8, and multidrug resistance (MDR) ovarian cancer cell lines SKOV-3TR and OVCAR8TR used in this study were characterized previously [45–48]. All cells were cultured in RPMI 1640 (Life Technologies, Grand Island, NY) supplemented with 10% fetal bovine serum (FBS) and 1% penicillin/streptomycin (Life Technologies, Carlsbad, CA) at 37°C in atmosphere composed of 5% CO_2_ and 95% air. Human non-specific siRNA and CD44 targeted endoribonuclease-prepared siRNA (esiRNA, Genebank Accession Number NM_001001391, 5′-TTAAAGGGATTCCCATCATTGGAATCTTAT-3′) were purchased from Sigma-Aldrich (St. Louis, MO). esiRNAs are synthesized by *in vitro* transcription of a 300–600 bp gene specific dsRNA, followed by enzymatic digestion using RNAses (i.e., RNase III). esiRNAs are pools of siRNAs that all target the same mRNA sequence with high specificity. This strategy eliminates the trial and error approach of identifying a useful synthetic siRNA and ensures minimal risk of off-target effects [49]. Therefore, we selected CD44 targeted esiRNA as transient transfection tool in this study. Lipofectamine^®^ RNAiMAX was purchased from Life Technologies Corp. The monoclonal mouse anti-human CD44 antibody was obtained from Cell Signaling Technology, which was produced by immunizing BALB/c mice with stimulated human leukocytes and recognizes residues surrounding Ser210 of human CD44. The monoclonal rabbit anti-human Pgp antibody, which recognizes endogenous levels of total MDR1/ABCB1 protein, was also obtained from Cell Signaling Technology.

### Establishment of stable CD44 knockdown ovarian cancer cell line by CD44 shRNA

Further validation of CD44 knockdown phenotype in ovarian cell line was carried out with CD44 lentiviral shRNA (Sigma-Aldrich). The shRNA sequence targeting CD44 corresponded to coding regions (5′-CCGGATGGACTCCAGTCATAGTATACTCGAGTA TACTATGACTGGAGTCCATTTTTTG-3′) of the CD44 gene. As compared with siRNA or esiRNA, which can transiently (usually between 24 to 96 hours) knockdown gene expression as described above, the advantage of using lentiviral shRNA is the ability to create a stable CD44 knockdown cell line. OVCAR8 expresses CD44 to some extent and is susceptible to the lentivirus-screening antibiotic puromycin. Hence, OVCAR8 was used to establish the stable CD44 knockdown ovarian cancer cell line in this study. Briefly, OVCAR8 cells were seeded (2 × 10^3^ per well) in a 96-well microplate and incubated with CD44 shRNA lentivirus for 24 hours at 37°C in a humidified incubator in an atmosphere of 5% CO_2_. Untreated cells were used as blank control; a lentiviral empty vector and lentivirus-based non-specific shRNA were used as negative controls. Each lentiviral construct and control was plated in duplicate wells. Hexadimethrine bromide was added to enhance transduction efficacy. Fresh medium containing 2 *μ*g/ml puromycin, which is lethal to most lentivirus-untransduced cells, was exchanged every 3–4 days until resistant colonies were identified (Figure 3 Panel A). The knockdown efficiency of CD44 shRNA was then confirmed by western blot when all cell lines grew vigorously. These cells lines were coined as OVCAR8^CD44 shRNA^, OVCAR8^Lentivirus only^, and OVCAR8^Non-specific shRNA^ according to the above-mentioned different treatments.

### Determination of the CD44 knockdown effect on spheroid formation in 3-Dimensional (3-D) culture of ovarian cancer cells

Cell spheroid formation was performed following HDP1096 Perfecta3D^®^ 96-Well Hanging Drop Plates Protocol (3D Biomatrix). Initially, due to the small volume of hanging drops, which makes them susceptible to evaporation and the variability in incubator conditions, pre-heated agarose solution was filled into the reservoirs of plate to better maintain the humidity condition of spheroid culture. Hanging drops were formed by pipetting 40 *μ*L of cell suspension (2.5 × 10^5^/ml) into each well, OVCAR8, OVCAR8^Lentivirus only^, OVCAR8^Non-specific shRNA^, and OVCAR8^CD44 shRNA^, respectively. 10 *μ*L of fresh medium was added back into the hanging drops slowly every other day to provide enough nutrients for cells and to prevent osmolality shift of the medium. The spheroids were harvested for further study from the bottom side of the plate by pipetting 100 *μ*L Phosphate Buffer Solution (PBS) into each well gently without any destruction of the morphology of the spheroids. Finally, the spheroids were photographed on a Nikon Eclipse Ti-U inverted fluorescence microscope (Nikon Instruments, Inc NY, CA) after incubating with 1 *μ*M Calcein AM (Life Technologies) for 15 min. The size of tumor spheroids was computed based on image analysis (by the software ImageJ).

### Cell viability analysis

Proliferation ability and cytotoxicity of anticancer drug paclitaxel in ovarian cancer cells were assessed by MTT (3(4,5-dimethylthiazol-2-yl)-2,5-diphenyl tetrazolium bromide) assay after CD44 was knocked down by CD44 shRNA. Well-established cells (OVCAR8^Lentivirus only^, OVCAR8^Non-specific shRNA^, as well as OVCAR8^CD44 shRNA^) were seeded into 96-well microplate at the density of 1 × 10^3^ cells per well and exposed to different concentrations of paclitaxel which were obtained from the pharmacy at the Massachusetts General Hospital. After 7-day incubation with a series concentrations of paclitaxel, 20 *μ*L MTT (Sigma-Aldrich) was added and then incubated for another 4 hours at 37°C and 5% CO_2_ humidified atmosphere. Subsequently, the resulting intracellular formazan crystals was solubilized in acid-isopropanol. The absorbances were assessed on a SpectraMax Microplate^®^ Spectrophotometer (Molecular Devices LLC, Sunnyvale, CA) at 490 nm, and normalized to the value of untreated cells.

### Western blot

Protein lysates of the cells and tissues were extracted with 1 × RIPA lysis buffer (Upstate Biotechnology, Charlottesville, VA) plus complete protease inhibitor cocktail tablets (Roche Applied Science, IN, USA). The protein concentrations were evaluated using Protein Assay Reagents (Bio-Rad, Hercules, USA) and a SPECTRAmax Microplate Spectrophotometer from Molecular Devices (Sunnyvale). Equal amounts of proteins were separated by NuPAGE^®^ 4–12% Bis-Tris Gel (Life Technologies), transferred onto nitrocellulose membrane (Bio-Rad), and incubated with specific primary antibodies (CD44 at 1: 1000 dilutions; Pgp at 1: 1000 dilutions; *β*-Actin at 1: 2000 dilutions) at 4°C overnight. The membranes were further probed with respective secondary antibodies (LI-COR Bioscences, Lincoln, NE), and scanned by Odyssey^®^ CLx equipment (LI-COR Bioscences) to detect the bands. Furthermore, the density of the bands was quantified by Odyssey software 3.0 (LI-COR Bioscences).

### Immunofluorescence

Immunofluorescence assay was utilized for visualizing the post transfection expression levels of CD44 in ovarian cancer cells. Firstly, the 48-hour post transfection cells were incubated in 4% paraformaldehyde, fixed in ice-cold methanol, and blocked with 1% bovine serum albumin (BSA). Then, immunostaining was performed using the CD44 antibody, Alexa Fluor 594 (Red) conjugated goat anti-mouse antibody (Life Technologies), and 1 *μ*g/ml Hoechst 33342 (Life Technologies) for counterstaining of nuclei. Finally, the cells were photographed on a Nikon Eclipse Ti-U fluorescence microscope (Nikon Instruments, Inc NY, CA) equipped with a SPOT RT™ digital camera. Red color represented CD44 protein and blue color represented cell nuclei.

### Wound healing assay

The effect of CD44 knockdown in ovarian cancer cells on migration was evaluated by wound healing assay. In brief, 2 × 10^5^ cells were seeded onto a 12-well plate, and transfected with CD44 esiRNA or non-specific siRNA. When the cells reached 80–100% confluence, three parallel lines with similar width were then created in each well using sterile 200 *μ*L pipette tips and rinsed to clear cell debris and suspension cells. Fresh regular RPMI 1640 medium was added and the cells were allowed to close the wound for 24 hours. Three images were captured per well at different time points after wounding (0, 8 and 24 hours) to monitor the repair process by a microscope (Nikon) at 100× magnification. The width of the wound was measured at 10 sites in each image. The cell migration distance was calculated by subtracting the wound width at each time point from the wound width at the 0 hour.

### Matrigel invasion assay

Matrigel invasion assay examined the alteration of cell invasion activity using a BD BioCoat™ Matrigel™ Invasion Chamber (Becton-Dickinson, MA) following the manufacturer's recommendations. Specifically, 5 × 10^4^ cells were added into the upper chamber of each well in FBS-free and antibiotics-free medium, and transfected with CD44 esiRNA or non-specific siRNA in the same transfection protocol as mentioned above, while the bottom chambers were filled with 500 *μ*L medium with 10% FBS without antibiotics. After a 48-hour incubation period, the non-invading cells were carefully scrubbed from the upper surface of the membrane with a cotton swab. Following the processes of fixation in 100% methanol and staining in hematoxylin, the invading cells were counted in three images of each membrane under a microscope using a 200× objective.

### Statistic analysis

The data were analyzed using Prism 5.0 software (Graph Pad Software Inc., San Diego, CA), and expressed as mean ± SEM. Statistical significance was assessed using independent two-tailed Student *t*-tests for independent data. Disease free survival and overall survival were analyzed using Kaplan-Meier survival curves with Gehan-Breslow-Wilcoxon test for significance. Differences of *P* < 0.05 were considered significant for all statistical tests.

## SUPPLEMENTARY TABLE


