# Supplementary material for: Up-regulation of CD44 in the development of metastasis, recurrence and drug resistance of ovarian cancer
Source: Oncotarget. 2015 Mar 13;6(11):9313–26. doi: 10.18632/oncotarget.3220 (PMC4496219; doi:10.18632/oncotarget.3220)
Supplement: Supplementary file 1 [file oncotarget-06-9313-s001.pdf]

SUPPLEMENTARY TABLE

Supplementary Table S1: Clinical Information of Ovarian Cancer Tissue Microarray

		Primary	Metastatic	Recurrent
**FIGO Stage**	III	15		
	IV	11		
**Grade**	1	1		
	2	4		
	3	21		
**Histological Types**	Serous	18	18	17
	Endometroid	1	1	1
	Transitional Cell	1	1	1
	Mixed Types	5	5	4
	Others/Unknown	1	1	3
**Ascites Present at Surgery**	Yes	17		
	No	9		
